# Wideband Spectrum Sensing Based on Riemannian Distance for Cognitive Radio Networks

**DOI:** 10.3390/s17040661

**Published:** 2017-03-23

**Authors:** Qiuyuan Lu, Shengzhi Yang, Fan Liu

**Affiliations:** School of Information and Electronics, Beijing Institute of Technology, Beijing 100081, China; adrien_yang@bit.edu.cn (S.Y.); liufan92@bit.edu.cn (F.L.)

**Keywords:** cognitive radio, wideband spectrum sensing, information geometry, Riemannian distance, Riemannian mean, moment matching

## Abstract

Detecting the signals of the primary users in the wideband spectrum is a key issue for cognitive radio networks. In this paper, we consider the multi-antenna based signal detection in a wideband spectrum scenario where the noise statistical characteristics are assumed to be unknown. We reason that the covariance matrices of the spectrum subbands have structural constraints and that they describe a manifold in the signal space. Thus, we propose a novel signal detection algorithm based on Riemannian distance and Riemannian mean which is different from the traditional eigenvalue-based detector (EBD) derived with the generalized likelihood ratio criterion. Using the moment matching method, we obtain the closed expression of the decision threshold. From the considered simulation settings, it is shown that the proposed Riemannian distance detector (RDD) has a better performance than the traditional EBD in wideband spectrum sensing.

## 1. Introduction

In today’s increasingly crowded wireless spectrum environment, cognitive radio (CR) networks are considered a promising technology to mitigate the contradiction between fixed spectrum allocation and efficient utilization, which has received sustained attention in recent years [1,2]. In order to access the spectrum holes without interfering with the primary user (PU) in the network, the secondary user (SU) is required to perform accurate spectrum sensing.

As a key problem in CR networks, spectrum sensing technology has been extensively studied. Several classical spectrum sensing methods, such as energy detection (ED), matched filtering (MF) detection and cyclostationary detection have been proposed. Although ED is easy to implement, it is sensitive to noise uncertainty [3]. The MF method requires waveform information about the primary signal [4] and cyclostationary detection needs to know the cyclic frequencies of the primary signal [5]. Compared with the classical methods, eigenvalue-based spectrum sensing methods for multi-antenna systems require less prior information about noise and signal. Due to the correlation of the primary signal in multi-antenna reception, some spectrum sensing algorithms are designed according to the covariance matrix of the received data vectors, and the corresponding test statistics are constructed by applying the eigenvalues of the covariance matrix. To date, some typical detectors have been proposed in the literature, including the largest eigenvalue detector (LED) [6], maximum-minimum eigenvalue (MME) detector [7], the scaled largest eigenvalue (SLE) detector [8], and the arithmetic to geometric mean (AGM) detector [9]. In practical application scenarios, the CR system may suffer from unknown interference in the wideband spectrum and the imperfections of the sensor hardware [10,11,12]. Thus the independent and identically distributed (i.i.d.) noise assumption in eigenvalue-based algorithms may not be realistic. Moreover, it is possible for a number of asynchronous independent PUs to access the same frequency band at the same time. Accordingly, the CR sensor needs to sense the wideband spectrum in the case of the unknown noise characteristics and unknown number of PUs.

Meanwhile, in CR networks, spectrum sensing for wideband is also an important topic worthy of study. In [13], the wideband spectrum sensing (WSS) problem is partitioned into four basic elements to study, namely, system modeling, performance metrics, sampling schemes and detection algorithms. In the context of this paper, we choose the detection probability of the primary signal in each subband of the wideband spectrum as the performance metric. We assume independent PU occupancy across subbands and that the detections of primary signals in each subband are independent with each other. For the above independent channel-by-channel detection mode [13], we can employ the more easily achieved partial band Nyquist sampling scheme to complete the wideband spectrum data acquisition.

In this paper, unlike traditional spectrum sensing algorithms based on a generalized likelihood ratio test (GLRT) [9,14], we consider the rethinking of WSS problem from the perspective of information geometry. As a promising cutting-edge discipline, information geometry studies the problems in the field of statistics and information science by applying modern differential geometry method on Riemannian manifolds. It has been widely used in machine learning, medical imaging, radar signal processing, signal classification and other research areas [15,16,17,18,19,20]. Inspired by these studies, we consider using the information geometry theory to design the spectrum sensing method, rather than the normed linear space theory. From the view of Lie group theory, the covariance matrix of sensing data is a Toeplitz Hermitian positive definite matrix, which forms the negative curvature space [21]. By constructing statistical models on the manifold, we can exploit the Riemannian distance for hypothesis testing, regardless of the specific characteristic assumptions about signal and noise. On the other hand, in multiband detection, there may be a plurality of vacant subbands available for estimating noise. Considering the geometric features of the matrix manifold, we use the Riemannian mean instead of the arithmetic mean for the joint estimation with multiple covariance matrices. To the best of the authors’ knowledge, this is the first time the information geometry is used to solve the WSS problem.

The rest of the paper is organized as follows: in Section 2, we describe the system model in detail, introduce the key concepts of information geometry—Riemannian distance and Riemannian mean, and propose the Riemannian distance-based test statistic. Section 3 presents theoretical analysis and finds thresholds for the proposed detector using random matrix theory. In Section 4, we show the numerical results of the proposed algorithm. Finally, the main results of this paper are drawn in Section 5.

## 2. System Model and the Proposed Detection Scheme

### 2.1. System Model

**Figure 1:**
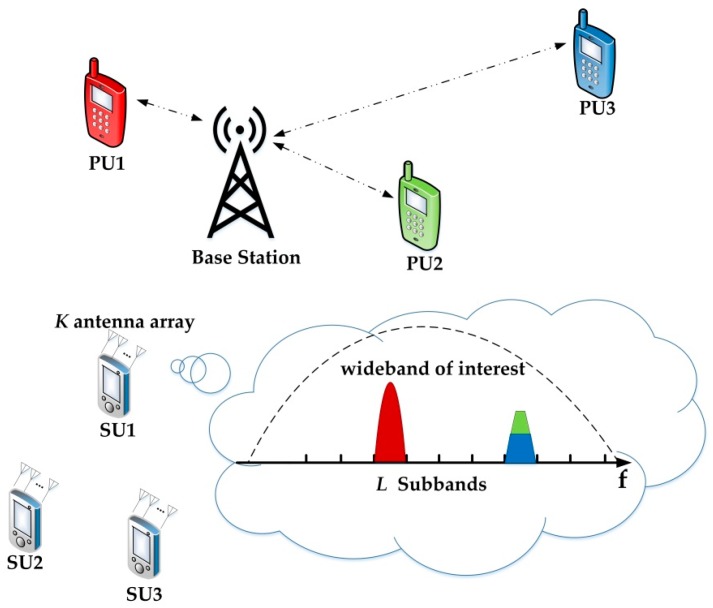
The CR system model with multiple PUs and *k* antenna SUs.

Consider a CR system performing spectrum sensing on a spectrum of *B* Hz equipped with *K* antennas in each sensing node, as depicted in Figure 1. In the context of this article, we focus on the spectrum sensing of a single SU. It is worth noting, however, that in the cooperative spectrum sensing model, our methods are still applicable if the data of different nodes can be collected at the fusion center. It is assumed that the bandwidths of subbands are known to the CR receiver and the whole band is divided into *L* subbands. In the sensing time $T_{s}$, the receiver collects *N* samples in each subbands, the *n*-th observed data in the *i*-th subband is $x_{i}[n],\;n=0,\ldots ,\;N-1$. Every subband sampling vector is composed of *K*-antenna data $x_{i}[n]={\left[x_{i}^{1}[n],\ldots ,x_{i}^{K}[n]\right]}^{T}$. Then, the spectrum sensing problem in each subband can be expressed by the following two hypotheses:

(1) $$\begin{array}{l} {ℋ}_{0,i}\;:x_{i}[n]=w_{i}[n],\;n=0,\ldots ,N-1\\ {ℋ}_{1,i}\;:x_{i}[n]={Η}_{i}s_{i}[n]+w_{i}[n],\;n=0,\ldots ,N-1, \end{array}$$

where ${ℋ}_{0,i}$ (null hypothesis) stands for the absence of PUs and ${ℋ}_{1,i}$ for the presence of PUs. Here, $w_{i}[n]$ is the *n*-th noise sample in the *i*-th subband over the *K* antennas. In our sensing scenario assumption, the number of concurrent PU transmissions is *P* which is unknown to the sensing node. The *K*×*P* matrix $H_{i}=[h_{i}^{\left(1\right)},\ldots ,h_{i}^{\left(P\right)}]$ represents the channels between the *P* PUs and the receiving antennas in the *i*-th subband. The *P*×1 vectors $s_{i}[n]={\left[s_{i}^{\left(1\right)}[n],\ldots ,s_{i}^{\left(P\right)}[n]\right]}^{T}$ denote the *n*-th samples of the transmitted signal from the PUs in the *i*-th subband. We assume that the transmitted samples $s_{i}^{\left(p\right)}[n]$ follow an i.i.d. zero mean complex Gaussian distribution, and are independent from the noise.

The entire collected observations of *i*-th subband is defined as a *K*×*N* matrix $X_{i}=[x_{i}[0],\ldots ,x_{i}[N-1]]$, where the noise matrix is the *K*×*N* matrix $W_{i}=[w_{i}[0],\ldots ,w_{i}[N-1]]$, and the signal matrix is the *P*×*N* matrix $S_{i}=[s_{i}[0],\ldots ,s_{i}[N-1]]$.

By the above assumptions, we define the covariance matrix of *i*-th subband as $R_{i}=E[x_{i}[n]x_{i}^{H}[n]]$, where $(\cdot {)}^{H}$ denotes the conjugate transpose. With *N* received samples in a finite sensing time, the CR receiver calculates the *K*×*K* sample covariance matrix ${\hat{R}}_{i}=X_{i}X_{i}{}^{H}$ to complete the sensing problem.

In our sensing problem assumption, the noise samples subject to zero mean complex Gaussian distribution, and the noise covariance matrix in *i*-th subband is assumed to be $\Psi_{i}$. Under the hypothesis ${ℋ}_{0,i}$, the sample covariance matrix is a complex Wishart matrix subject to $W_{K}(N,\Psi_{i})$. Under the hypothesis *ℋ*_1,*i*_, the sample covariance matrix is denoted as:

(2) $${ℋ}_{0,i}:{\hat{R}}_{i}=W_{i}W_{i}{}^{H}=\Psi_{i}$$

(3) $${ℋ}_{1,i}:{\hat{R}}_{i}=H_{i}S_{i}S_{i}{}^{H}H_{i}{}^{H}+\Psi_{i}.$$

With the uncorrelated assumption about $S_{i}$, and the transmission power of the *p*-th PU defined as $\eta_{i}^{\left(p\right)}=E(s_{i}^{\left(p\right)}[n]s_{i}^{\left(p\right)}{\left[n\right]}^{H})$, Equation (3) can be written as:

(4) $${\hat{R}}_{i}=\sum_{p=1}^{P}\eta_{i}^{\left(p\right)}h_{i}^{\left(p\right)}h_{i}^{\left(p\right)}{}^{H}+\Psi_{i}.$$

The sensing performance is evaluated by the detection probability $P_{D}$ and the false alarm probability $P_{F}$, respectively, corresponding to the correct detection of the presence of primary signal at hypothesis ${ℋ}_{1}$ and wrongly claiming of the presence of primary signal at hypothesis ${ℋ}_{0}$.

### 2.2. Riemannian Distance and Riemannian Mean

In information geometry theory, we consider that ℱ is a set of probability density functions p(x|θ), where x is a sample of *n* dimensional complex random variable X, i.e., **x** ∈ **X** ⊆ *C^n^*. And θ is the *m* dimensional parameter vector, i.e., **θ** ∈ Θ ⊆ *C^m^*. Generally, the probability distribution space can be described by its parameter set Θ. The statistical model S is expressed as:

(5) $$S=\{p(x|\theta )|\theta \in \Theta \subseteq C^{m}\},$$

Under certain topological structures, S can form a differentiable manifold, called statistical manifold ℳ, where θ is defined as the natural coordinate of the statistical manifold [22]. From the point of view of information geometry, the probability distribution $W_{K}(N,\hat{R_{i}})$ can be parameterized separately by the respective covariance matrix. Then $W_{K}(N,\hat{R_{i}})$ can be considered to be located on the statistical manifold which takes covariance matrix as coordinate. In particular, ${\hat{R}}_{i}$ is a Toeplitz Hermitian positive definite matrix with the noise and signal models under ${ℋ}_{0}$ and ${ℋ}_{1}$. The set of covariance matrices constitutes a complex symmetric positive definite (SPD) matrix space denoted by Sym(n,C), which is also defined as SPD manifold. The parameter space Θ of zero-mean multivariate Gaussian distribution and Sym(n,C) are isomorphic. Therefore the statistical manifold can be described by Sym(n,C) due to the mapping relationship between them [23]. Many articles have focused on the research of the geometry structure of complex symmetric positive definite matrix manifold. It is a completely connected, complete Riemannian manifold with non-positive sectional curvature, called the Cartan-Hadamard manifold, whose geodesic exists and is unique [21]. In all curves connecting the two points $\theta_{A}\theta_{B}$ on the manifold, the geodesic is the shortest one. This shortest distance, called the Riemannian distance between $\theta_{A}$ and $\theta_{B}$, can be used to describe the similarity of the two distributions. The Riemannian distance between two elements in Sym(n,C) is given in [19] as follows:

(6) $$\begin{array}{ll} D^{2}\left(R_{1},R_{2}\right) & ={\left\|\log\left(R_{1}^{-1/2}R_{2}R_{1}^{-1/2}\right)\right\|}^{2}\\ & ={\left\|\log\left(R_{1}^{-1}R_{2}\right)\right\|}^{2}\\ & =Tr[\log^{2}\left(R_{1}^{-1}R_{2}\right)]\\ & =\sum_{i=1}^{n}\log^{2}\left(\lambda_{i}\right)\;, \end{array}$$

where ∥⋅∥ is the Frobenius norm and *λ_i_* is the *n* eigenvalues of the matrix $R_{1}^{-1}R_{2}$. Compared with Kullback-Leibler divergence, $D^{2}$ has better properties, such as symmetry, satisfying triangle inequality and so on.

Let $\bar{R}$ be the midpoint between two points $R_{1}$ and $R_{2}$ in space. In the normed linear space, the midpoint calculation corresponds to the arithmetic mean $\bar{R}=\left(R_{1}+R_{2}\right)/2$. On the manifold, it is found that for Sym(n,C), the local curvature is not constant Sym(n,C), the local curvature is not constant and not positive, so the calculation of the midpoint must depend on the corresponding geometric mean rather than the arithmetic mean. Using the Riemannian distance we can define the midpoint satisfying $D^{2}\left(R_{1},\bar{R}\right)=D^{2}\left(R_{2},\bar{R}\right)$. The formula for calculating the midpoint with the geometric means is given as $\bar{R}=R_{1}^{1/2}{\left(R_{1}^{-1/2}R_{2}^{1/2}R_{1}^{-1/2}\right)}^{1/2}R_{1}^{1/2}$ in [19]. For the *N* points on Sym(n,C), the Riemannian mean is defined by Riemannian distance:

(7) $$\bar{R}=\arg\min_{R\in Sym(n,C)}\frac{1}{N}\sum_{k=1}^{N}D^{2}(R_{k},R).$$

**Figure 2:**
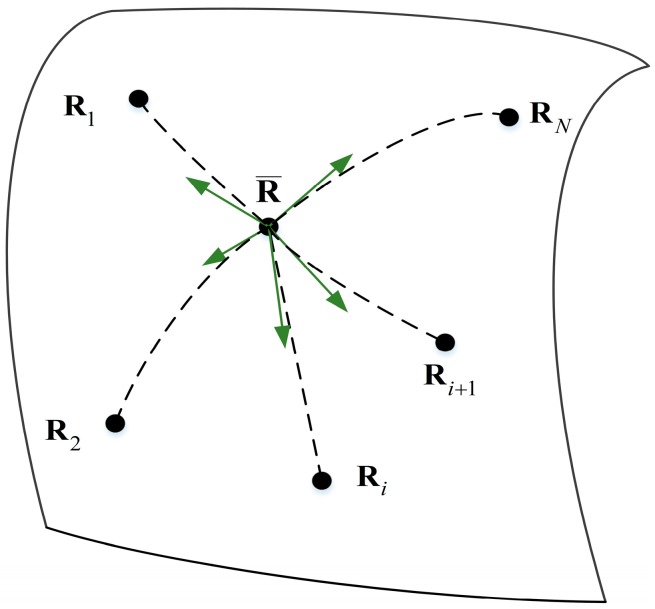
The Riemannian mean of *N* points on the manifold.

The geometric representation of the Riemann mean is given in Figure 2. In the field of information geometry research, Riemannian mean computation is an important problem. In [17], a Riemannian mean computation method based on the gradient descent algorithm is proposed. The Riemannian mean is iteratively computed by using the gradient descent algorithm along the geodesic direction of the manifold:

(8) $${\bar{R}}_{t+1}={\bar{R}}_{t}^{1/2}e^{-\varepsilon \sum_{k=1}^{N}\log\left({\bar{R}}_{t}^{-1/2}R_{k}^{1/2}{\bar{R}}_{t}^{-1/2}\right)}{\bar{R}}_{t}^{1/2}.$$

In (8), ε≥0 controls the iteration speed and $R_{k}\left(k=1,2,\ldots ,N\right)$ are the *N* matrices on the manifold. ${\bar{R}}_{t}$ is the estimate of the Riemannian mean calculated from *t* iterations. The detailed process of iterative computation is given in Algorithm 1.
**Algorithm 1:** Iterative Calculation of the Riemannian Mean By a Gradient Descent Algorithm**Input**: $R_{k}\left(k=1,2,\ldots ,N\right)$ and ε.**Output**: Estimates of Riemannian mean $\bar{R}$.**Initialize**: t=1; ${\bar{R}}_{1}=R_{1}$.**repeat**Compute gradient of objective function $\nabla f=\sum_{k=1}^{N}\log\left({\bar{R}}_{t}^{-1/2}R_{k}^{1/2}{\bar{R}}_{t}^{-1/2}\right)$;Obtain ${\bar{R}}_{t+1}={\bar{R}}_{t}^{1/2}e^{-\varepsilon \nabla f}{\bar{R}}_{t}^{1/2}$;Update t=t+1;**until** convergence.

### 2.3. The Riemannian Distance Based Test Statistic

In our sensing scenario assumptions, the specific form of $\Psi_{i}$ is arbitrary and unknown, but the $\Psi_{i}$ of different subbands are identical. This assumption of noise means the different subbands suffer from the same unknown interference or the imperfections of the sensor hardware. It is difficult to design the test statistic according to the probability distributions of the received data under ${ℋ}_{0}$ under such assumptions. We consider the sample covariance matrix of the vacant channel in the wideband spectrum as an estimate of the unknown $\Psi_{i}$, called the reference matrix. Then we calculate the Riemannian distance between the covariance matrix of the channel under test and the reference matrix. Thus, the solution to the detection problem is transformed from the traditional statistical inference method (which is accomplished by hypothesis testing the probability distribution of the statistical model via the *N* observations of X) into the information geometry method (which is accomplished by computing the geodesic between the two points on manifold).

There are several ways to get the reference matrix. One is to extract noise-only data samples in the system's agreed free time and frequency bands. If the interval between the free time and the sensing time is short enough, or the free bands is close enough to the bands under test, they can be considered to have the same noise covariance characteristics. Besides, we can select one or more subbands with the lower power spectral density (PSD) as the vacant noise-only subbands as detailed in [24]. The sample covariance matrix of the vacant subband is set to be the reference matrix for the Riemann distance based detection method.

If the reference matrix is ${\hat{R}}_{V}$, then the test statistic is defined as:

(9) $$\begin{array}{l} T_{RD}={\left\|\log\left({\hat{R}}_{V}^{-1/2}{\hat{R}}_{i}{\hat{R}}_{V}^{-1/2}\right)\right\|}^{2}\\ ={\left\|\log\left({\hat{R}}_{V}^{-1}{\hat{R}}_{i}\right)\right\|}^{2}\\ =\sum_{k=1}^{K}\log^{2}\left(\lambda_{k}\right)\;, \end{array}$$

where ${\hat{R}}_{i}$ is the *K*×*K* sample covariance matrix of the subband to be tested, and $\lambda_{k}\left(k=1,\ldots ,K\right)$ is the eigenvalue of the matrix ${\hat{R}}_{V}^{-1}{\hat{R}}_{i}$. The proposed Riemannian distance detector is:

(10) $$T_{RD}\overset{{ℋ}_{0,i}\;}{\underset{{ℋ}_{1,i}}{≶}}\gamma ,$$

where γ is the pre-set detection threshold.

In summary, we propose the process of spectrum sensing algorithm based on Riemannian distance in wideband:

1. Determine the vacant subband. If there is only one vacant (noise-only) subband, then its sample covariance matrix $\Psi_{V}=X_{V}{X_{V}}^{H}$ is calculated and used as a reference matrix ${\hat{R}}_{V}=\Psi_{V}$. If there are multiple vacant subbands, such as *A*, then the Riemannian mean ${\bar{\Psi }}_{V}$ of the noise covariance matrices $\Psi_{i}\left(i=1,\ldots ,A\right)$ of the multiple vacant subbands can be used as the reference matrix:
  (11) $${\hat{R}}_{V}={\bar{\Psi }}_{V}=\arg\min_{\Psi \in Sym(n,C)}\frac{1}{A}\sum_{i=1}^{A}D^{2}(\Psi_{i},\Psi ).$$
2. Compute the sample covariance matrix ${\hat{R}}_{i}=X_{i}X_{i}{}^{H}$ of the *i*-th subband to be tested.
3. Obtain the test statistic $T_{RD,i-\mathrm{th}\mathrm{subband}}=D^{2}({\hat{R}}_{V},{\hat{R}}_{i})=\sum_{k=1}^{K}\log^{2}(\lambda_{k})$, where $\lambda_{k}$ is the eigenvalue of ${\hat{R}}_{V}^{-1}{\hat{R}}_{i}$ with ordered $0\leq \lambda_{1}\leq \cdots \lambda_{K}\leq \infty$.
4. Compare the test statistic with the threshold, and get the sensing result:
  (12) $$T_{RD,i-th\;subband}\overset{{ℋ}_{0,i}\;}{\underset{{ℋ}_{1,i}\;}{≶}}\gamma .$$

In the above sensing method, the main computational complexity lies in the covariance matrix inversion calculation. The difficulty of inversion depends on the value of *K*, which is the number of antennas. As we will present in the Section 4, even with small *K*, the proposed Riemannian distance detector still shows good detection performance. If *K* is large, the matrix inversion calculation may not be easy. Then the alternative methods noted in [19] may be a better choice. It should be noticed that in Equation (6), we can calculate the square root matrix instead of the inverse matrix. In [19], a square root of positive definite matrix computing method is given. Using the Schulz iteration, the intractable matrix inversion can be avoided.

## 3. Threshold and Probability of False Alarm

The $P_{F}$ performance and $P_{D}$ performance of the proposed Riemannian distance detector depend on the probability distribution of the test statistic Equation (9) under ${ℋ}_{0}$ and ${ℋ}_{1}$. However, the analytical forms of the probability distribution under both hypotheses are hard to be obtained. In this section we derive the accurate closed-form approximation of $P_{F}$ to get the decision threshold. And the $P_{D}$ performance will be presented and discussed through numerical simulations in Section 4.

### 3.1. Moments of Test Statistics under ${ℋ}_{0}$

We consider a moment matching method to approximate the probability density function (PDF) of the test statistics. Therefore, we need to obtain the exact moments of $T_{RD}$ under ${ℋ}_{0}$:

(13) $$E\left(T_{RD}{}^{p}\right)=E\left\{{\left(\sum_{k=1}^{K}\log^{2}\left(\lambda_{k}\right)\right)}^{p}\right\}.$$

In this section, we will give a detailed calculation process of the moments of test statistics under ${ℋ}_{0}$. First, we show the joint PDF of the eigenvalues in Equation (9) according to the random matrix theory. Then we use the joint PDF to solve the moments of test statistics which can be regarded as the eigenvalue function. The calculation of the first order moments will be presented as an example. And the closed-form solution to the *p*-th moment will be given at last.

Consider the case where the number of reference matrices is *A* = 1, then the reference covariance matrix and the sample covariance matrix of the *i*-th subband in the expression of the test statistic $T_{RD}$ under ${ℋ}_{0}$ follow the distributions respectively:

(14) $${\hat{R}}_{V}\sim W_{K}(N,\Psi_{v}),$$

(15) $${\hat{R}}_{V}\sim W_{K}(N,\Psi_{v}),\;\mathrm{under}\;{ℋ}_{0},$$

where ${\hat{R}}_{V}$ is independent of ${\hat{R}}_{i}$. The joint PDF of the eigenvalues $\lambda_{k}\left(k=1,\ldots ,N\right)$ of the matrix ${\hat{R}}_{V}^{-1}{\hat{R}}_{i}$ has been given under the assumption that the dimensions of two complex Wishart matrices are equal [25,26], which is:

(16) $$f\left(\lambda_{1},\lambda_{2},\ldots ,\lambda_{K}\right)=\frac{\Gamma_{N}\left(2K\right)}{\Gamma_{N}\left(K\right)\Gamma_{N}\left(K\right)\Gamma_{N}\left(N\right)}\prod_{k=1}^{K}\frac{\lambda_{k}^{N-K}}{{\left(1+\lambda_{k}\right)}^{2K}}\prod_{k<i}^{K}{\left(\lambda_{i}-\lambda_{k}\right)}^{2}.$$

In the case of a known joint probability density of $\lambda_{k}$, we can refer to the moment calculation method in [27] to solve the moments of the eigenvalue function $T\left(\lambda_{1},\lambda_{2},\ldots ,\lambda_{K}\right)=\sum_{k=1}^{K}\log^{2}\left(\lambda_{k}\right)$. In general, if the joint probability density (16) can be written as:

(17) $$f\left(\lambda \right)=C_{0}\left|\Phi \left(\lambda \right)\right|\left|\Psi \left(\lambda \right)\right|\prod_{k=1}^{K}\xi \left(\lambda_{k}\right).$$

Then the expectation of the function $\beta \left(\lambda_{k}\right)$ for $\lambda_{k}$ can be solved as follows:

(18) $$E\{\prod_{k=1}^{K}\beta \left(\lambda_{k}\right)\}=C_{0}|U|,$$

where Φ(λ), Ψ(λ), U are *K*×*K* matrices. The (i,j) elements in Φ(λ) and **Ψ**(**λ**) are Φ_*i*_(*λ_j_* and $\Psi_{i}\left(\lambda_{j}\right)$, respectively. The (i,j) element in U can be expressed as:

(19) $$u_{i,j}=\int_{0}^{\infty }\Phi_{i}\left(\lambda \right)\Psi_{j}\left(\lambda \right)\xi \left(\lambda \right)\beta \left(\lambda \right)d\lambda .$$

By introducing the function $\omega \left(x\right)=e^{sx}$, we can use Equations (17) and (18) to solve the required moments:

(20) $$E\{e^{s\sum_{k=1}^{K}\log^{2}(\lambda_{k})}\}=E\{\prod_{k=1}^{K}e^{s\log^{2}(\lambda_{k})}\}.$$

By p-th order derivatives of (20) at s=0, we have the *p*-th moment of $T\left(\lambda_{1},\lambda_{2},\ldots ,\lambda_{K}\right)$:

(21) $$E\{{\left(\sum_{k=1}^{K}\log^{2}(\lambda_{k})\right)}^{p}\}=\frac{d^{p}}{d_{s}{}^{p}}\{E(e^{s{-}_{k=1}^{K}\log^{2}(\lambda_{k})})\}|{}_{s=0}.$$

We take $C_{0}=\frac{\Gamma_{N}\left(2K\right)}{\Gamma_{N}\left(K\right)\Gamma_{N}\left(K\right)\Gamma_{N}\left(N\right)}$ as the normalized coefficients determined by *N*, *K*, and choose Φ(λ)=Ψ(λ)=V(λ), where V(λ) is a Vandermonde matrix. Choosing $\xi \left(\lambda_{k}\right)=\frac{\lambda_{k}^{N-K}}{{\left(1+\lambda_{k}\right)}^{2K}}$, $\beta \left(\lambda_{k}\right)=e^{s\log^{2}\left(\lambda_{k}\right)}$, we can get:

(22) $$E\{\prod_{k=1}^{K}e^{s\log^{2}(\lambda_{k})}\}=E\{\prod_{k=1}^{K}\beta (\lambda_{k})\}=C_{0}|U(s)|.$$

The element $u{\left(s\right)}_{i,j}$ in matrix U(s) corresponds to the sub-item in (19) as follows that:

(23) $$\begin{array}{l} \Phi_{i}\left(\lambda \right)=V_{i}\left(\lambda \right)=\lambda^{i-1},\\ \Psi_{j}\left(\lambda \right)=V_{j}\left(\lambda \right)=\lambda^{j-1},\\ \xi \left(\lambda \right)=\frac{\lambda^{N-K}}{{\left(1+\lambda \right)}^{2N}},\\ \beta \left(\lambda \right)=e^{s\log^{2}\left(\lambda \right)}. \end{array}$$

Then (19) can be written as:

(24) $$u{\left(s\right)}_{i,j}=\int_{0}^{\infty }\frac{\lambda^{N-K+i+j-2}}{{\left(1+\lambda \right)}^{2N}}e^{s\log^{2}\left(\lambda \right)}d\lambda .$$

For the differential operation in (22), we can use the rules for the matrix determinant in [28]:

(25) $$\frac{d}{ds}\left|U\left(s\right)\right|=\sum_{k=1}^{K}\left|U_{\left(k\right)}\left(s\right)\right|,$$

where $U_{\left(k\right)}\left(s\right)$ is the matrix that coincides with U(s) except that every entry in the k-th row (equivalently, columns could be used) is differentiated with respect to *s*.

Then we take the first moment calculation as an example, for *p* = 1:

(26) $$E(T_{RD})=\frac{d}{ds}\{C_{0}\left|U\left(s\right)\right|\}|{}_{S=0}=C_{0}\sum_{k=1}^{K}|U_{\left(k\right)}\left(0\right)|.$$

$u{\left(0\right)}_{i,j}$ and $\frac{d}{ds}u{\left(s\right)}_{i,j}|{}_{s=0}$ are the two types of matrix elements in $U_{\left(k\right)}\left(0\right)$:

(27) $$u{\left(0\right)}_{i,j}=\int_{0}^{\infty }\frac{\lambda^{N-K+i+j-2}}{{\left(1+\lambda \right)}^{2N}}d\lambda =B\left(N-K+i+j-1,N+K-i-j+1\right),$$

(28) $$\frac{d}{ds}u{\left(s\right)}_{i,j}|{}_{s=0}=\int_{0}^{\infty }\frac{\lambda^{N-K+i+j-2}}{{\left(1+\lambda \right)}^{2N}}\log^{2}\left(\lambda \right)d\lambda ,$$

where B(x,y) is the Beta function. Thus the (i,j) element of $U_{\left(k\right)}\left(0\right)$ can be denoted as and the function $I_{k,j}\left(x\right)$ is defined by:

(29) $${\left[U_{\left(k\right)}\left(0\right)\right]}_{i,j}=\int_{0}^{\infty }\frac{\lambda^{N-K+i+j-2}}{{\left(1+\lambda \right)}^{2N}}I_{k,j}\left(\log^{2}\left(\lambda \right)\right)d\lambda .$$

(30) $$I_{k,j}\left(x\right)≜\left\{ \begin{array}{ll} x, & \mathrm{if}\;k=j\\ 1\;, & \mathrm{if}\;k\neq j \end{array}\right..$$

Similarly, the result of *p*-th moment can be obtained. There will be an integral term with the following form when computing the *p*-th moment:

(31) $$\frac{d^{p}}{ds^{p}}u{\left(s\right)}_{i,j}|{}_{s=0}=\int_{0}^{\infty }\frac{\lambda^{N-K+i+j-2}}{{\left(1+\lambda \right)}^{2N}}\log^{2p}\left(\lambda \right)d\lambda .$$

In Appendix A we give a detailed derivation for the integral form such as $\int_{0}^{\infty }\frac{t^{x-1}}{{\left(1+t\right)}^{x+y}}\log^{q}\left(t\right)dt$. To sum up, we can get the exact analytical *p*-th moment.

### 3.2. Gamma Approximation Approach

With the exact analytical *p*-th moment obtained in Section 3.1, we can use the gamma distribution function to approximate the test statistic under ${ℋ}_{0}$ according to the moment matching method in [29].

Using the gamma approximation, we only need to compute the first and second moments to obtain the mean and variance of the test statistic as follows:

(32) $$\begin{array}{l} \mu_{T}\overset{∆}{=}E(T_{RD})=M(1)\\ \sigma_{T}^{2}\overset{∆}{=}E(T_{RD}^{2})-{\left[E(T_{RD})\right]}^{2}=M(2)-M{\left(1\right)}^{2}, \end{array}$$

where M(p) is the *p*-th moment of $T_{RD}$. Suppose the test statistic satisfies the gamma distribution with shape parameter $k_{T}$ and scale parameter $\theta_{T}$ and $F_{T_{RD}}$ is denoted as the cumulative distribution function (CDF) of $T_{RD}$. According to the CDF of a gamma distribution defined in [30], $F_{T_{RD}}$ is derived as:

(33) $$\begin{array}{l} k_{T}=\frac{\mu_{T}^{2}}{\sigma_{T}^{2}}=\frac{M{\left(1\right)}^{2}}{M\left(2\right)-M{\left(1\right)}^{2}}\\ \theta_{T}=\frac{\sigma_{T}^{2}}{\mu_{T}}=\frac{M\left(2\right)-M{\left(1\right)}^{2}}{M\left(1\right)}, \end{array}$$

(34) $$F_{T_{RD}}\left(x\;;k_{T},\theta_{T}\right)=1-\frac{\Gamma \left(k_{T},x/\theta_{T}\right)}{\Gamma \left(k_{T}\right)},$$

where $\Gamma \left(k_{T},x/\theta_{T}\right)$ is the upper incomplete gamma function.

Denoting γ as the decision threshold, then the probability of false alarm under ${ℋ}_{0}$ is:

(35) $$P_{F}=1-F_{T_{RD}}\left(\gamma \;;k_{T},\theta_{T}\right).$$

So we can use the inverse of the gamma cumulative distribution function to compute the decision threshold:

(36) $$\gamma =F_{T_{RD}}^{-1}\left(1-P_{F}\;;k_{T},\theta_{T}\right).$$

Although the threshold calculation method we derived seems to be complex enough, such complexity may not be a serious problem in the spectrum sensing process. As we can see in the deduction process above, the threshold depends on the desired $P_{F}$ and the system parameters *N*, *K*. These parameters should be predefined in the CR node. Therefore threshold calculation does not need to be real-time. Even if the system is required to operate under different parameter set, we can still calculate the threshold of different parameter sets in advance and make a query table for real-time mode changing.

## 4. Numerical Results

In this section, we give the performance evaluation of the proposed algorithms by means of Monte Carlo simulations. First, we examine the accuracy of the $P_{F}$ approximation method and the decision threshold calculation. Then we compare the detection performance of RDD with some conventional EBDs in different scenarios. In addition, we show the performance of RDD when Riemannian mean method is applied in multiple vacant subbands situation. In this section of the simulation, we choose the value of *N*, *K* according to the practical system. Due to the limited sensing time and sampling rate, *N* generally takes tens to hundreds, while *K* is generally less than eight because of hardware size. We choose the exponential correlation model [31] as the noise covariance matrix:

(37) $${\left(\Psi_{0}\right)}_{i,j}=\rho^{\left|i-j\right|},\;\rho \in \left[0,1\right)$$

where ρ denotes the degree of noise correlation. And each subband has the same noise covariance matrix, i.e., $\Psi_{i}=\Psi_{0}$ for i=1,2,…,L.

In addition, we assume that the channel matrix $H_{i}$ of the subband is composed of independent Gaussian random variables and satisfies the normalization conditions ${\left\|H_{i}\right\|}^{2}=1$. In each Monte Carlo realization, the channel matrix is generated randomly. The PU signals in the simulation follow the i.i.d. zero mean complex Gaussian distribution, and are independent from the noise.

The received SNR of *p*-th primary signal in the *i*-th subband is defined as:

(38) $$SNR_{i}^{\left(p\right)}=\frac{\eta_{i}^{\left(p\right)}{\left\|H_{i}\right\|}^{2}}{\nu_{i}}$$

where $\eta_{i}^{\left(p\right)}$ is the transmission power of the *p*-th PU, and $\nu_{i}$ is the noise power in the *i*-th subband, which satisfies $\nu_{i}=Tr\left(\Psi_{i}\right)/K$. In the case of multiple PUs, we set different SNRs for different PUs due to the fact that the distance between multiple PUs and SU are not the same in the practical sensing situations.

### 4.1. Decision Threshold and P_F_

In Table 1, we present the numerical simulation of the moments computing method proposed in Section 3.1. It is worth noting that the joint PDF of the eigenvalues defined by (16) holds for any value of the covariance matrix parameter Σ in two complex Wishart distributions $W_{K}$(*N*,Σ). Hence, in the simulation, we can specify some combinations of the sample size *N* and antenna number *K*, and then generate the complex Wishart matrix with arbitrary covariance matrix to calculate the test statistic under ${ℋ}_{0}$. In Table 1, the accuracy of the theoretical calculation is verified by comparing the simulated and theoretical values of the first and second moment of the test statistic $T_{RD}$ under ${ℋ}_{0}$.

**Figure 3:**
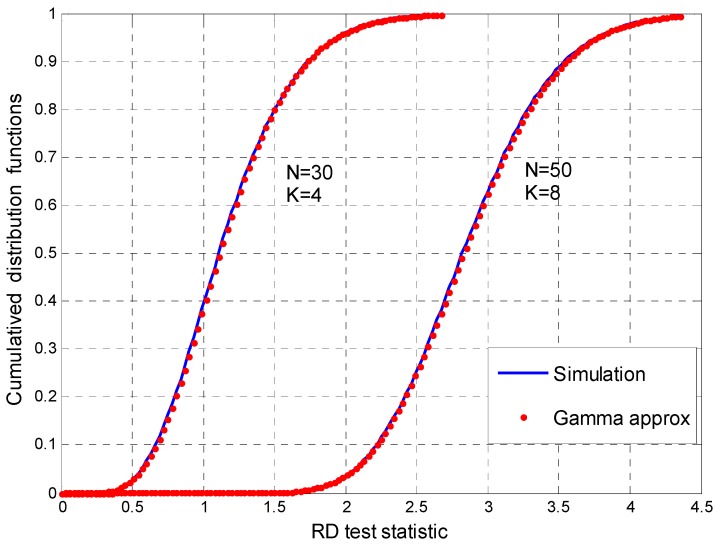
Cumulative distribution functions (CDFs) of the test statistics for (*N*,*K*) = (30,4) and (*N*,*K*) = (50,8).

In Figure 3, we give the simulation values of the CDF of the test statistic and the theoretical value of the gamma distribution approximation at the specified (*N*,*K*). This figure shows that the gamma distribution approximation based on the moment matching method proposed in Section 3.2 achieves good performance. Moreover, our approximation algorithm are perfectly matched to the simulation values for different (*N*,*K*).

**Figure 4:**
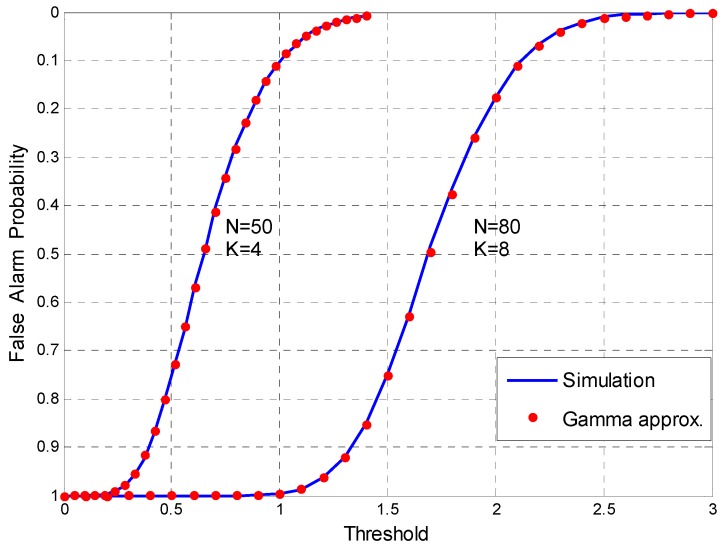
Decision threshold γ vs. $P_{F}$ for (*N*,*K*) = (50,4) and (*N*,*K*) = (80,8).

We plot the decision threshold as a function of $P_{F}$ for the specified (*N*,*K*) in Figure 4. The simulation value curve is obtained by Monte Carlo method. Meanwhile, the theoretical value of *P_F_* can be obtained directly by (35). The figure shows the perfect agreement between the theoretical computing and simulation results. Therefore, the sensing algorithm can get the corresponding decision threshold under the specified (*N*,*K*) and the desired $P_{F}$. In this way, the detector can satisfy the constant false alarm rate (CFAR) requirements of the CR system.

### 4.2. Detection Performance

First, the Receiver Operating Characteristic (ROC) curve is used to compare the performance of the proposed detector and other detectors when the number of reference matrices is *A* = 1. The ROC curve gives the detection probability $P_{D}$ as a function of the false alarm probability $P_{F}$. By changing the threshold γ, the operating point of the detector can be chosen anywhere along its ROC curve.

Considering that the noise variance, noise covariance matrix and the number of PUs are both unknown in our assumption, blind detection is necessary. Among the conventional EBDs, the SLE detector and the AGM detector are two typical blind detectors [32]. According to the classification of EBD in [29], they correspond to arithmetic mean detector (ARMD) and arithmetic-geometric mean detector (AR-GEMD), respectively. Both detectors operate in a single-band spectrum sensing scenario and do not require noise-only subband for detection. In order to deploy these two EBDs in multi-band detection, we use the pre-whitening technique [33] to improve their sensing performance in multi-band with unknown noise. By replacing ${\hat{R}}_{i}$ by ${\hat{R}}_{V}^{-1}{\hat{R}}_{i}$ for the EBD in the *i*-th subband, we can compare the performance of EBD and the proposed RDD in the simulation.

**Figure 5:**
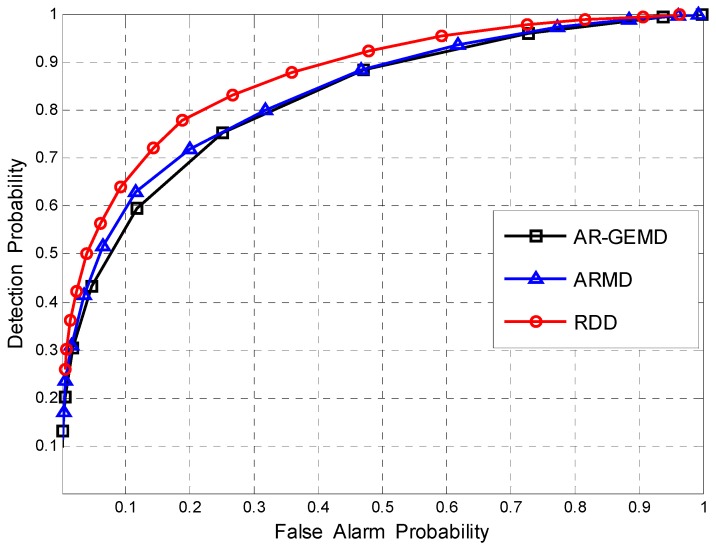
The ROCs comparison for (*N*,*K*) = (100,4). Assuming *P* = 1, ρ=0.4, and $SNR^{\left(1\right)}=-3dB$.

The ROC curves of the detectors when there is one primary signal are plotted in Figure 5 where $SNR^{\left(1\right)}=-3dB$, ρ=0.4 and (*N*,*K*) = (100,4). It is clear that the proposed detector outperforms the two EBDs under the assumption of correlated noise.

**Figure 6:**
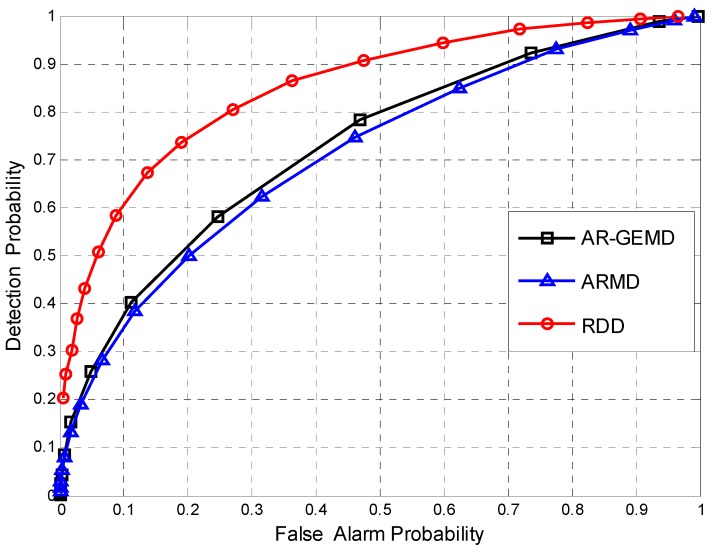
The ROCs comparison for (*N*,*K*) = (100,4). Assuming *P* = 3, ρ=0.4, and $SNR^{\left(1\right)}=-6dB$.

**Figure 7:**
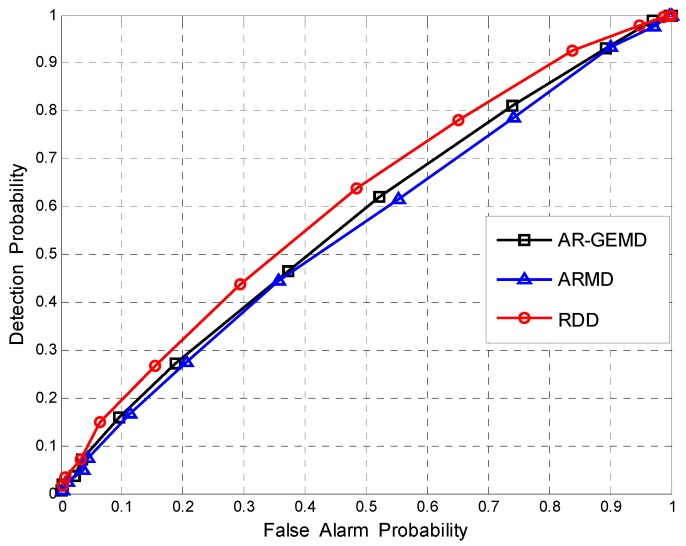
The ROCs comparison for (*N*,*K*) = (80,8). Assuming *P* = 5, ρ=0.4, and $SNR^{\left(1\right)}=-8dB$.

Figure 6 and Figure 7 show the ROC curves when the number of PUs is assumed to be *P* = 3 and *P* = 5 respectively. The SNRs of multiple PUs are defined as $SNR^{\left(i+1\right)}=SNR^{\left(i\right)}-1dB$. In the case of different combinations of *N* and *K*, we can see the same performance differences between RDD and EBDs as in Figure 5. It should be seen that ARMD performs better than AR-GEMD in the case of single PU but worse in the case of multiple PUs. As noted in [9,14], the rank of received covariance matrix is assumed in the derivation of the GLR-based detector. In our signal model, the number of PUs has an effect on the rank of the covariance matrix, resulting in the EBD method being selective to *P*, as shown in the figures. By contrast, RDD is not sensitive to the number of PUs because we do not make any assumptions about *P* in the proposed algorithm. From the geometric point of view, the detection performance of RDD depends on the Riemannian distance between ${\hat{R}}_{i}$ and ${\hat{R}}_{V}$. Although the combination of different primary signals will lead to the position change of ${\hat{R}}_{i}$ on the manifold, the distance-based detection method remains effective.

**Figure 8:**
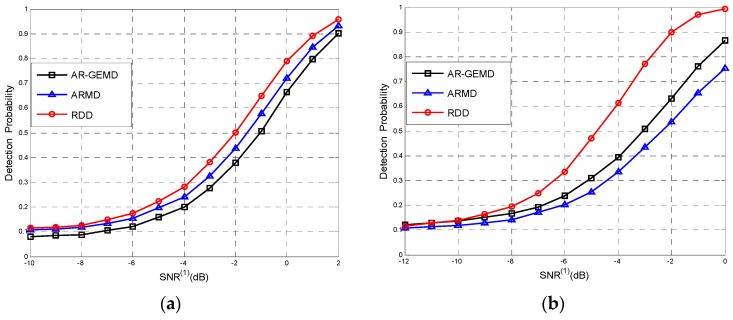
The probability of detection versus *SNR* for (*N*,*K*) = (50,4) and ρ=0.4 in different PU numbers (**a**) *P* = 1; (**b**) *P* = 3.

In practical, communication systems often require a constant $P_{F}$, as required by the IEEE 802.22 standard to be 10% [34]. We plot $P_{D}$ at different average received *SNR* in Figure 8 and choose (*N*,*K*) = (50,4) and ρ=0.4, while the decision threshold has been set to achieve $P_{F}=0.1$. And the number of PUs is one in Figure 8a and three in Figure 8b. The figures illustrate that the RDD can achieve higher detection probability than EBD for the same *SNR* level.

### 4.3. Multiband Detection with Riemannian Mean

In this section, we consider the performance of RDD when multiple vacant subbands are present, i.e., *A* > 1. First, we would like to evaluate the performance of matrix mean estimation between the Riemannian mean (RM) method and the arithmetic mean (AM) method. In the simulation, the matrix dimension parameter is (*N*,*K*) = (80,8), the matrix type is the correlated noise matrix, and the correlation coefficient ρ satisfying the uniform distribution U(0,1) is randomly generated in each Monte Carlo implementation. The objective function in the Riemannian mean expression (7), which is the mean distance, can be written as follows:

(39) $$f\left(R_{k},R_{t}\right)=\frac{1}{A}\sum_{k=1}^{A}D^{2}\left(R_{k},R_{t}\right)\;.$$

**Figure 9:**
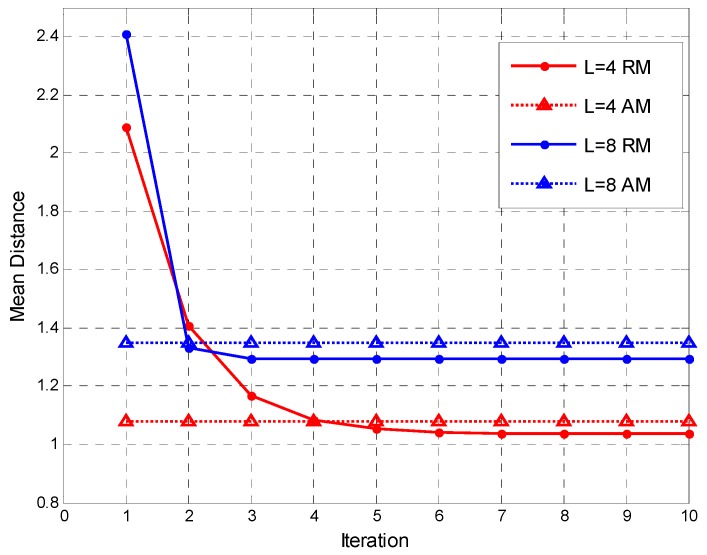
Estimation Performance Comparison for (*N*,*K*) = (80,8) and random ρ when the number of reference matrices is *A* = 4 and *A* = 8.

The trend of the mean distance with the iteration number *t* is shown in Figure 9. When calculating (8), we choose *ε* = 0.1 to control the iteration speed. Meanwhile, in Figure 9 we show the comparison of RM and AM estimation performance when the number of reference matrices is *A* = 4 and *A* = 8. It can be observed that the objective function of the RM calculation based on the gradient descent algorithm decreases as the number of iterations increases, and tends to be stable after a certain number of iterations. It should be noted that the iterative computation is not required in AM method, so the mean distance of AM in the figure remains unchanged. The figure shows that for different number of reference matrices, the mean distance of the converged RM method is less than that of the AM method.

**Figure 10:**
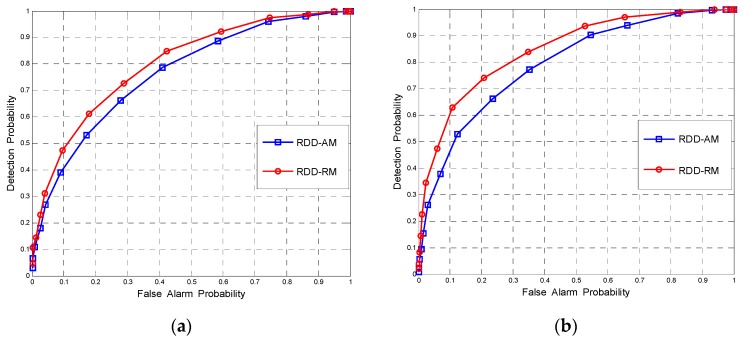
The ROCs of RDD using AM method and RM method for (*N*,*K*) = (80,8), *ρ* = 0.4, *P* = 3 and *SNR*^(1)^ = −5 with different number of reference matrices (**a**) *A* = 2; (**b**) *A* = 4.

Finally, we compare the ROC curves of RM method and the AM method in the case of multiple vacant subbands. We choose (*N*,*K*) = (80,8), *ρ* = 0.4, *P* = 3 and *SNR^(1)^* = −5 for the simulation. Figure 10a,b plot the ROC curves with reference matrices *A* = 2 and *A* = 4, respectively, where the iteration number of RM method is 8. We can see that for RDD, RM estimation outperforms the AM estimation, and when the number of reference matrices is large, the gap is more obvious. The above simulation results illustrate that the sensing performance can be improved if the RM matrix of reference matrices is used for the RDD when multiple vacant subbands are available in WSS.

## 5. Conclusions

In this paper, we propose a novel WSS detector based on Riemannian distance for multi-antenna CR. For a wideband spectrum divided into several subbands, we compute the Riemannian distance of covariance matrices between the vacant subband and the subband to be detected. Through theoretical analysis, we obtain the exact closed expression of the decision threshold using the moment matching method. Unlike the traditional EBD derived with the GLR criterion, the proposed RDD is derived from information geometry theory. By applying the geometric method, we do not have to make too many assumptions about noise and primary signals like the traditional methods do. Therefore we obtain a detector which is blind to noise statistical characteristics and number of PUs. The simulation results show that the proposed detector exhibits better performance than the conventional EBD method in the correlated noise model and is robust to the number of PUs. Moreover, we propose a matrix mean estimation method based on RM. In the presence of multiple vacant subbands, the RM method can better estimate the noise distribution than the AM method. Thus it is more suitable for RDD due to the better use of the wideband spectrum information for sensing.

## Figures and Tables

**Table 1:** Numerical results of $T_{RD}$.

(N,K)	E(*T_RD_*) Simulated	E(*T_RD_*) Analytical	$E(T_{\mathrm{RD}}^{2})$ Simulated	$E(T_{\mathrm{RD}}^{2})$ Analytical
(30,4)	1.1708	1.1695	1.5489	1.5474
(50,4)	0.6750	0.6756	0.5147	0.5151
(50,8)	2.8708	2.8667	8.5091	8.4914
(80,8)	1.7135	1.7424	3.1125	3.14

## Appendix A

According to the definition of Beta function [30] we have:

(A1) $$B\left(x,y\right)=\int_{0}^{\infty }\frac{t^{x-1}}{{\left(1+t\right)}^{x+y}}dt,$$

(A2) $$\frac{\partial^{q}}{\partial x^{q}}B\left(x,y\right)=\int_{0}^{\infty }\frac{t^{x-1}}{{\left(1+t\right)}^{x+y}}\log^{q}\left(\frac{t}{1+t}\right)dt,$$

(A3) $$\frac{\partial^{q}}{\partial y^{q}}B\left(x,y\right)=\int_{0}^{\infty }\frac{t^{x-1}}{{\left(1+t\right)}^{x+y}}{\left[-\log\left(1+t\right)\right]}^{q}dt.$$

By transformation as:

(A4) $${\left\{\log\left(\frac{t}{1+t}\right)-\left[-\log\left(1+t\right)\right]\right\}}^{q}=\log^{q}t$$

We can easily obtain:

(A5) $$\int_{0}^{\infty }\frac{t^{x-1}}{{\left(1+t\right)}^{x+y}}\log^{q}tdt={\left(\frac{\partial }{\partial x}-\frac{\partial }{\partial y}\right)}^{q}B\left(x,y\right)\;.$$

In calculating the first and second moments of the test statistic, there are q=2 and q=4 in the integral equation to be solved. They can be expressed as follows:

(A6) $$\int_{0}^{\infty }\frac{t^{x-1}}{{\left(1+t\right)}^{x+y}}\log^{2}tdt=B_{2,0}\left(x,y\right)+B_{0,2}\left(x,y\right)-2B_{1,1}\left(x,y\right),$$

(A7) $$\int_{0}^{\infty }\frac{t^{x-1}}{{\left(1+t\right)}^{x+y}}\log^{4}tdt=B_{4,0}\left(x,y\right)+B_{0,4}\left(x,y\right)-4B_{3,1}\left(x,y\right)-4B_{1,3}\left(x,y\right)+6B_{2,2}\left(x,y\right)$$

where $\frac{\partial^{m}}{\partial x^{m}}\frac{\partial^{n}}{\partial y^{n}}B\left(x,y\right)=B_{m,n}\left(x,y\right)$. The required $B_{m,n}\left(x,y\right)$ in the computing can be written as a combination of digamma functions, such as:

(A8) $$B_{1,0}\left(x,y\right)=B\left(x,y\right)\left(\psi \left(x\right)-\psi \left(x+y\right)\right),$$

(A9) $$B_{2,0}\left(x,y\right)=B\left(x,y\right)\left(\psi \left(1,x\right)-\psi \left(1,x+y\right)+{\left(\psi \left(x\right)-\psi \left(x+y\right)\right)}^{2}\right).$$

Similarly, we can derive other $B_{m,n}\left(x,y\right)$ using digamma function expansion.
